# Reply to ‘Comment on “Coherent control in the extreme ultraviolet and attosecond regime by synchrotron radiation”’

**DOI:** 10.1038/s41467-021-24029-4

**Published:** 2021-06-18

**Authors:** Y. Hikosaka, T. Kaneyasu, M. Fujimoto, H. Iwayama, M. Katoh

**Affiliations:** 1 Institute of Liberal Arts and Sciences, University of Toyama, Toyama, Japan; 2 SAGA Light Source, Tosu, Japan; 3 Institute for Molecular Science, Okazaki, Japan; 4 Sokendai (The Graduate University for Advanced Studies), Okazaki, Japan; 5 Hiroshima Synchrotron Radiation Center, Hiroshima University, Higashi-Hiroshima, Japan

**Keywords:** Atom optics, Optical spectroscopy

**Replying to** Prince et al. *Nature Communications* 10.1038/s41467-021-24024-9 (2021)

We recently demonstrated the capability of synchrotron radiation to perform coherent control in the extreme ultraviolet and attosecond regime^1^. This was demonstrated by implementing wave-packet interferometry^2–4^ on Rydberg wave packets generated in helium atoms. Prince and Diviacco performed a similar (but fundamentally different) experiment using a tandem undulator and argue that our work does not provide evidence of achieving coherent control with synchrotron radiation^5^. They state that the light from a tandem undulator is not a double pulse, but a single pulse with duration corresponding to the electron bunch length (typically hundreds of ps). This single light pulse consists of light-wave packets emitted by individual relativistic electrons randomly distributed within the electron bunch, with poor temporal coherence among the light-wave packets. They claim that coherent control is not possible by using such incoherent light pulses, since coherent control always requires a coherent light source. In contrast, we have shown that wave-packet interferometry can indeed be achieved, making use of the temporal coherence not among the different light-wave packets, but within individual (double-pulse) light-wave packets.

Relativistic electrons traveling through a tandem undulator radiate light-wave packets having a common form defined by the magnetic field of the undulator. Figure 1a presents calculated temporal profiles of light wave packets emitted from a single relativistic electron, under three different conditions of the phase shifter installed between the two undulators. The profiles were calculated using the synchrotron radiation calculation code SPECTRA^6^, using the nominal machine parameters of the storage ring we utilized in our work^1^. The calculation shows that the light-wave packet is of double-pulse shape, and that the time separation between the two components can be adjusted by changing the conditions of the phase shifter. Applying the Fourier transformation to the temporal profiles of the light-wave packets provides the spectra in the frequency domain (Fig. 1b). The derived spectra show fringed structures, with the number of fringes increasing with increasing time separation between the two components of the light-wave packet. Prince and Diviacco present essentially similar fringe-like spectra measured with their tandem undulator (Fig. 2 in ref. ^5^). The observation of fringes in their spectra confirms the fact that individual light-wave packets are of double-pulse shape with well-defined phase relation, both in our setup and that of Prince and Diviacco.

**Fig. 1:**
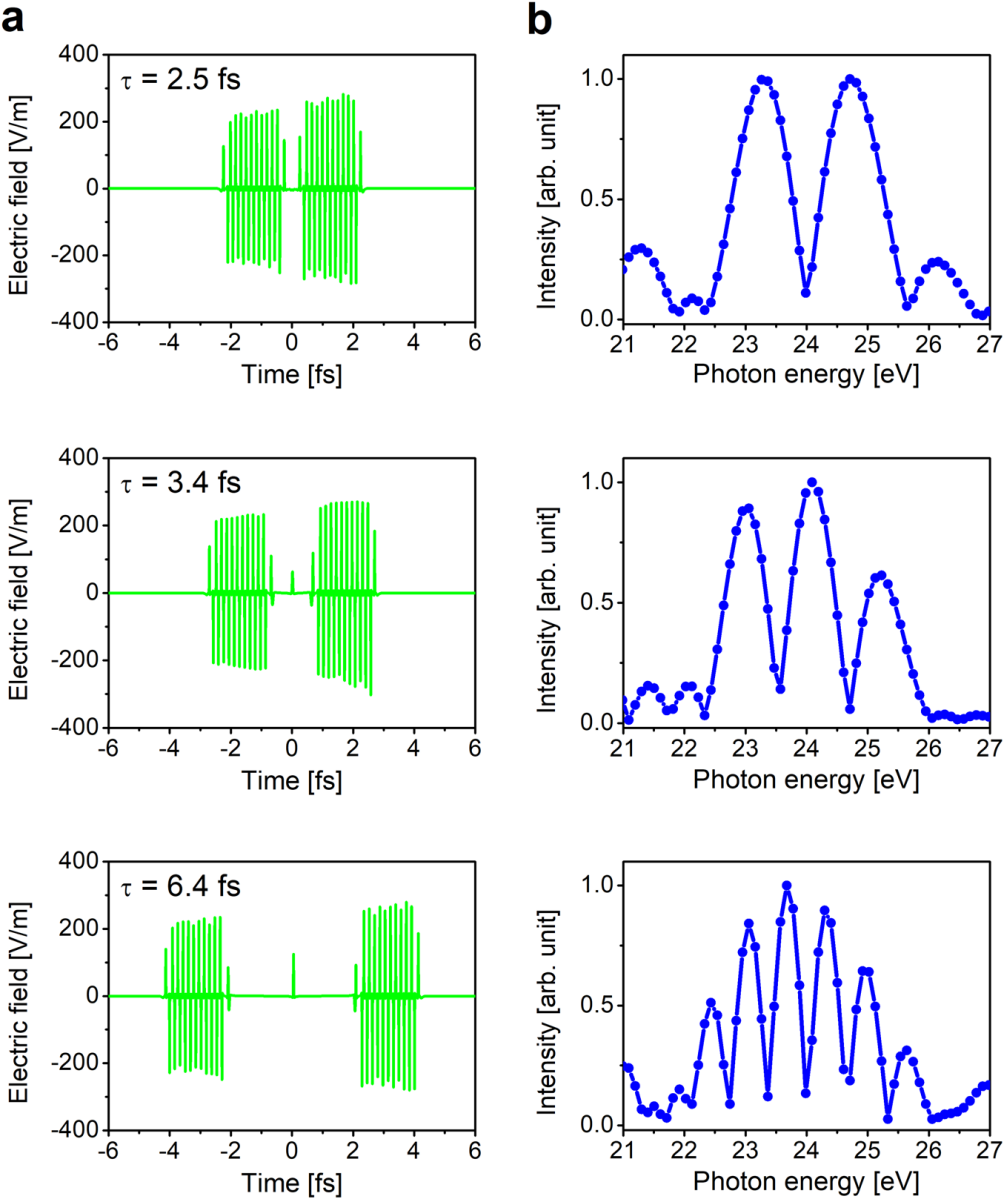
Radiation from a relativistic electron traveling through a tandem undulator. **a** Temporal profiles of a light-wave packet for three different conditions of the phase shifter installed between the two undulators. These profiles were calculated with the synchrotron radiation calculation code SPECTRA^5^, using the nominal machine parameters of the UVSOR storage ring. The time delay τ formed by the phase shifter is noted in each panel. **b** Spectra of the radiation from the tandem undulator, obtained by applying the Fourier transformation to the temporal profiles shown in (**a**).

When a light-wave packet with a double-pulse shape coherently excites several resonant states in an atom or a molecule, two coherent wave packets are generated in the target^7^. In practical measurement, the target is exposed to radiation from a bunch of electrons, and thus successively interacts with numerous light-wave packets sharing a common profile. As we have already discussed in some detail^8^, interactions of a single target with random light-wave packets cancel each other out, and what remains is the result of the interaction with a single (double-pulse) light-wave packet, in which the relative phase of the two components is locked. This basis is common to previous observations of Ramsey interferences induced with non-transform-limited laser pulses^9,10^.

By tuning the time delay between the double-pulse components of the light-wave packet, one can adjust the interference of the two wave packets generated in the target and achieve population control of the excited states^11^. This is because the time-domain Ramsay fringes seen in the time evolutions of the excited state populations oscillate with distinct resonant frequencies. The population control can be described in the frequency domain as being due to differences in intensity at the wavelengths corresponding to different resonances—a delay can be chosen to enhance the population of the desired state and suppress those of other states. This correspondence between the time-domain and frequency-domain pictures of wave-packet interferometry is widely known^12^.

In Fig. 1 of ref. ^5^, Prince and Diviacco showed that the monochromatic intensity of undulator radiation changes as a function of the phase shifter condition, and the photoemission yield of their sample is just proportional to the monochromatic flux irradiated. Their observation clarifies that the intensity at a particular wavelength varies as a function of the phase shifter, with a modulation frequency that corresponds to the wavelength. This is the essential requirement for population control, as discussed above.

We believe that the reason why Prince and Diviacco present arguments based on the monochromatic intensity of the undulator radiation is due to not considering the difference between the concept of optical interferences between the two light pulses and the concept of quantum interferences between electronic wave packets. Optical interferences occur when two light pulses overlap temporally within the coherence length. Considering that the coherence length of each of the sequential radiations from our tandem undulator is around 2 fs, and the sequential radiations are naturally separated by the electron delay (see Fig. 1a), the light from the tandem undulator does not show optical interference as it is. In contrast, the temporal oscillations in the monochromatic intensity observed by Prince and Diviacco (Fig. 1 of ref. ^5^) result from monochromatization, which greatly increases the coherence length of the light and allowing optical interference to occur. On the other hand, the Ramsey fringe presented in Fig. 3a of ref. ^1^ results from the quantum interference between two Rydberg electron wave packets launched by the projection of the light-wave packet of double-pulse shape, and this quantum interference occurs beyond the coherence length of light. Very recently, with this wave-packet interferometry scheme, we succeeded in tracking the femtosecond Auger decays of Xe inner-shell excited states in the time domain^13^.

Prince and Diviacco draw an analogy between our method using a tandem undulator and Fourier Transform spectrometry. While Fourier Transform spectrometry is usually utilized as spectroscopy of quantum states, wave-packet interferometry is a method to control populations and pathways of quantum states by the interference of wave packets generated in the matter. In our work, two Rydberg electron wave packets are generated in helium by the projection of the double-pulse structures of a light-wave packet, and the interference between the coherent wave packets is utilized to control the populations of the Rydberg states.

Finally, we comment on the limitation of coherent control with synchrotron radiation, as it seems that Prince and Diviacco see the present scheme as the Tannor-Rice pump-dump scheme^14^. In the latter scheme, the first light pulse produces a single wave packet usually on an excited potential surface of a molecule and the second light pulse dumps the wave packet to another potential surface, after the travel of the wave packet to a particular point to promote the desired reaction pathway. The pump-dump scheme relies on two-photon processes that cannot be realized with the small-amplitude wave packets of synchrotron radiation.

In conclusion, we believe our work shows that un-monochromatized synchrotron light from a tandem undulator does indeed consist of light-wave packets of double-pulse shape, and that the temporal coherence within the individual light-wave packets can be utilized for implementing wave-packet interferometry. The observations of Prince and Diviacco do not show that population control cannot be realized with synchrotron radiation, and actually clarify the essence of the experimental scheme.

## Data Availability

The data that support the findings of this study are available from the corresponding author upon reasonable request.

## References

[1] Hikosaka Y, Kaneyasu T, Fujimoto M, Iwayama H, Katoh M (2019). Coherent control in the extreme ultraviolet and attosecond regime by synchrotron radiation. Nat. Comm..

[2] Bouchene MA (1998). Temporal coherent control induced by wave packet interferences in one and two photon atomic transitions. Eur. Phys. J. D..

[3] Blanchet V (1998). Temporal coherent control in the photoionization of CS2: theory and experiment. J. Chem. Phys..

[4] Ohmori K (2009). Wave-packet and coherent control dynamics. Annu. Rev. Phys. Chem..

[5] Prince KC, Diviacco B (2019). On “Coherent control in the extreme ultraviolet and attosecond regime by synchrotron radiation” by Hikosaka et al. Nat. Comm..

[6] Tanaka T, Kitamura H (2001). SPECTRA: a synchrotron radiation calculation code. J. Synchrotron Radiat..

[7] Noordam LD, Duncan DI, Gallagher TF (1992). Ramsey fringes in atomic Rydberg wave packets. Phys. Rev. A.

[8] Kaneyasu T, Hikosaka Y, Fujimoto M, Iwayama H, Katoh M (2020). Polarization control in a crossed undulator without a monochromator. N. J. Phys..

[9] Jones RR, Schumacher DW, Gallagher TF, Bucksbaum PH (1995). Bound-state interferometry using incoherent light. J. Phys. B: . Mol. Opt. Phys..

[10] Snoek LC, Clement SG, Harren FJM, van der Zande WJ (1996). Femtosecond interferometric photoacoustic spectroscopy using incoherent light. Chem. Phys. Lett..

[11] Ohmori K, Sato Y, Nikitin EE, Rice SA (2003). High-precision molecular wave-packet interferometry with HgAr dimers. Phys. Rev. Lett..

[12] Dantus M, Lozovoy V (2004). Experimental coherent laser control of physicochemical processes. Chem. Rev..

[13] Kaneyasu T, Hikosaka Y, Fujimoto M, Iwayama H, Katoh M (2021). Electron wave packet interference in atomic inner-shell excitation. Phys. Rev. Lett..

[14] Tannor DJ, Kosloff R, Rice SA (1986). Coherent pulse sequence induced control of selectivity of reactions: exact quantum mechanical calculations. J. Chem. Phys..

